# Human Mesenchymal Stem Cells Modified with the NS5A Gene of Hepatitis C Virus Induce a Cellular Immune Response Exceeding the Response to DNA Immunization with This Gene

**DOI:** 10.3390/biology12060792

**Published:** 2023-05-30

**Authors:** Olga V. Masalova, Ekaterina I. Lesnova, Vladimir A. Kalsin, Regina R. Klimova, Natalya E. Fedorova, Vyacheslav V. Kozlov, Natalya A. Demidova, Kirill I. Yurlov, Mikhail A. Konoplyannikov, Tatyana N. Nikolaeva, Alexander V. Pronin, Vladimir P. Baklaushev, Alla A. Kushch

**Affiliations:** 1 Gamaleya National Research Center for Epidemiology and Microbiology, Ministry of Health of the Russian Federation, 123098 Moscow, Russia; wolf252006@yandex.ru (E.I.L.); regi.k@mail.ru (R.R.K.); ninani@mail.ru (N.E.F.); hyperslava@yandex.ru (V.V.K.); ailande@yandex.ru (N.A.D.); kir34292@yandex.ru (K.I.Y.); tatyananik.55@mail.ru (T.N.N.); proninalexander@yandex.ru (A.V.P.); vitallku@mail.ru (A.A.K.); 2 Federal Research Clinical Center of Specialized Medical Care and Medical Technologies, Federal Medical-Biological Agency of the Russian Federation, 115682 Moscow, Russia; vkalsin@mail.ru (V.A.K.); mkonopl@mail.ru (M.A.K.); baklaushev.vp@fnkc-fmba.ru (V.P.B.); 3 Institute for Regenerative Medicine, Sechenov First Moscow State Medical University, 119435 Moscow, Russia

**Keywords:** hepatitis C virus (HCV), human mesenchymal stem cells (MSCs), genetically modified MSCs, DNA immunization, non-structural HCV NS5A protein, cellular immune response, HCV vaccine, memory T cells, myeloid derived suppressor cells (MDSCs)

## Abstract

**Simple Summary:**

Hepatitis C is an infectious disease caused by the hepatitis C virus (HCV). Chronic viral hepatitis C is asymptomatic for many years and can lead to the development of liver cirrhosis/cancer. A vaccine is needed to eliminate hepatitis C; however, numerous attempts to create vaccines have so far been unsuccessful. The aim of this work was to investigate the ability of genetically modified human mesenchymal stem cells (mMSCs) expressing the HCV NS5A protein to induce a cellular immune response to HCV. Sixteen stem cell lines of a various origin were tested, and MSCs obtained from the dental pulp were selected which most effectively expressed the HCV protein. The triple immunization of mice with mMSCs showed an increase in the antigen-specific lymphocyte proliferation, the greater production of antiviral cytokine interferon-gamma, as well as an increase in the number of CD4+ memory T cells compared with the immunization with the NS5A gene. Thus, these results, which were obtained for the first time, show that human mMSCs can be a basis for the development of an effective vaccine against hepatitis C that triggers a strong T cell response and prevents the transition of acute hepatitis C to the chronic form due to the rapid activation of memory cells.

**Abstract:**

Hepatitis C virus (HCV) is one of the basic culprits behind chronic liver disease, which may result in cirrhosis and hepatocarcinoma. In spite of the extensive research conducted, a vaccine against HCV has not been yet created. We have obtained human mesenchymal stem cells (hMSCs) and used them for expressing the HCV NS5A protein as a model vaccination platform. Sixteen hMSC lines of a different origin were transfected with the pcNS5A-GFP plasmid to obtain genetically modified MSCs (mMSCs). The highest efficiency was obtained by the transfection of dental pulp MSCs. C57BL/6 mice were immunized intravenously with mMSCs, and the immune response was compared with the response to the pcNS5A-GFP plasmid, which was injected intramuscularly. It was shown that the antigen-specific lymphocyte proliferation and the number of IFN-γ-synthesizing cells were two to three times higher after the mMSC immunization compared to the DNA immunization. In addition, mMSCs induced more CD4+ memory T cells and an increase in the CD4+/CD8+ ratio. The results suggest that the immunostimulatory effect of mMSCs is associated with the switch of MSCs to the pro-inflammatory phenotype and a decrease in the proportion of myeloid derived suppressor cells. Thus, the possibility of using human mMSCs for the creation of a vaccine against HCV has been shown for the first time.

## 1. Introduction

Hepatitis C virus (HCV) is one of the basic culprits behind chronic liver diseases worldwide. There are more than 71 million infected people in the world, and 3–4 million are infected annually. About 400,000 patients die every year from the terminal stages of chronic hepatitis C (CHC)—liver cirrhosis and hepatocarcinoma [1]. Up to 80% of cases of acute hepatitis C turn into a chronic form, which is explained by the increased frequency of HCV mutations, the interference of viral proteins with the factors of innate and adaptive host immunity, and the formation of “escape” variants of HCV that elude the immune response [2]. Despite the advent of potent direct antiviral agents (DAA) based on HCV enzyme inhibitors—non-structural NS3 and NS5B proteins, as well as NS5A protein blockers, the extremely high cost makes the treatment inaccessible. In addition, the long-term effects of blocking viral enzymes, as well as the occult type of hepatitis C, which involves the HCV RNA present in hepatocytes or peripheral blood mononuclear cells (PBMCs) and is absent in the serum, have not been sufficiently studied [3]. Some side effects of the treatment with DAA are already known, such as HCV mutations, relapses, and reinfections [4,5]. The recent studies have shown that the disappearance of the virus after DAA does not lead to the complete normalization of immune cell function: the proliferation of T cells is not restored, the synthesis of proinflammatory and profibrous cytokines continues, and the expansion of suppressor cells is observed [6,7]. A successful elimination of HCV with DAA cannot provide protection against reinfection.

The lack of a vaccine is the main obstacle in the control of hepatitis C. The basis for the development of vaccines is presented by mainly genetically engineered products that mimic HCV sequences: recombinant proteins, peptides representing B- and T-cell epitopes, and DNA nucleotide sequences in plasmids and viral vectors [8,9]. However, the optimal composition of HCV genes or proteins (or their parts) for inclusion in the vaccine has not been determined. Non-structural proteins of the HCV replicative complex play a critical role in the cellular antiviral immune response. Thus, in spontaneously recovered patients with acute hepatitis C, CD4+ (T-helper, Th) and CD8+ (cytotoxic, CTL) lymphocytes reacting with the epitopes of NS3, NS4A, NS4B, NS5A and NS5B proteins were detected, which were not detected in patients with CHC. In addition, non-structural proteins, unlike the variable surface proteins E1 and E2, have highly conserved regions within their structure, with some T-cell epitopes localized there [10,11]. A high HCV-specific response to the T-cell epitopes of non-structural proteins can limit infection, eliminate HCV-infected cells, and prevent the development of acute hepatitis. In this regard, non–structural proteins are promising candidates for the inclusion in the vaccine.

The non-structural NS5A protein is a multifunctional phosphoprotein. It plays a crucial role in viral pathogenesis and in the induction of immune reactions, and also performs a number of important functions in the viral life cycle. This protein can be a transcription activator, and is involved in many cellular regulatory processes. NS5A, in addition to directly participating in virus replication, plays a key role in the early stages of the assembly and maturation of viral particles [12,13]. NS5A is associated with the modulation of the response to interferon (IFN) treatment; in particular, HCV resistance to IFN-α correlates with the number of mutations in NS5A [14]. A number of modern pangenotypic DAAs (Ledipasvir, Pibrentasvir, Velpatasvir, etc.) are aimed at blocking the functions of NS5A, and an active search is underway for new inhibitors of this protein [15]. Currently, a large number of studies are being conducted that are aimed at developing vaccine preparations for the creation of hepatitis C vaccines, but none of the candidate vaccines has yet been able to provide full-fledged preventive and therapeutic effects against HCV [8,9,16].

Mesenchymal stem cells (MSCs) have been efficiently applied in various fields of regenerative medicine [17]. MSCs are immunoprivileged cells, and their administration does not lead to an inflammatory immune response. MSCs can be harvested from almost all tissues, such as bone marrow, adipose tissue, umbilical cord blood, dental pulp, and the periosteal jaw. Cell therapy is based on MSCs’ ability to direct migration to lesions, and on their anti-inflammatory and immunomodulatory effects in allogeneic transplantation, as well as in autoimmune diseases [18,19,20,21]. The genetic modification of MSCs (mMSCs) providing the expression of certain introduced genes essentially boosts the potential of both cell and gene therapy, with therapeutic molecules delivered directly to the sites of injury and inflammation [22,23]. The immunosuppressive effects of MSCs have been well studied, but there is practically no data on the ability of human MSCs and mMSCs to induce an immune response.

We have previously shown that immunizing mice with mMSCs expressing a complex of non-structural NS3-NS5B HCV proteins significantly increases the immune response compared to a plasmid carrying the same genes [24]. MSCs were obtained from the bone marrow of syngenic mice. However, the properties of human MSCs differ from those of mouse MSCs [25,26]. The purpose of this study was to investigate the ability of human mMSCs carrying the NS5A gene to induce a cellular immune response against HCV.

## 2. Materials and Methods

### 2.1. Human Mesenchymal Stem Cells (MSCs)

This study was approved by the ethics committee of the Federal Research Clinical Center of Specialized Medical Care and Medical Technologies, Federal Medical-Biological Agency of the Russian Federation (FRCC FMBA) of Moscow. All of the study participants provided informed consent prior to their inclusion in the study. All of the experiments were in accordance with the Declaration of Helsinki, and were approved by the Ethics Committee of the FRCC FMBA (No. 2, 29 April 2018). Human samples were obtained from healthy adults from the dental pulp (bone marrow and adipose tissue) of clinically healthy exfoliated deciduous teeth or maternal cord blood under protocols approved by the ethical review board at the respective institutions. Bone marrow derived (BM), umbilical cord blood derived (UC), adipose tissue derived (AD), and dental pulp derived (DP) MSCs (BM-MSCs, AD-MSCs, UC-MSCs, and DP-MSCs, respectively) were isolated as previously described [27,28,29,30]. The cells’ cultural and morphological properties, the expression of surface receptors, and the ability to differentiate were characterized. The adhesive ability, multipotency with osteogenic, chondrogenic and adipogenic potentials, and the expression of a characteristic set of surface markers verify their mesenchymal origin and meet the minimum criteria for determining the MSCs of the International Society for Cellular Therapy [31]. Cells were cultured at 37 °C in 5% CO_2_ in the DMEM/F-12 growth medium with a 10% fetal bovine serum (FBS) (Gibco, Walthman, MA, USA), 100.0 U/mL penicillin, and 100.0 µg/mL streptomycin (PanEco, Moscow, Russia), or in a serum-free α-MEM medium with the addition of the human platelet lysate (HPL) (Merck, St. Louis, MO, USA) as described in Table 1. Cells at early passages were frozen and stored in liquid nitrogen. The FetMSC cell line from the embryonic bone marrow was obtained from the collection of cell cultures of the Institute of Cytology of the Russian Academy of Sciences, St. Petersburg, Russia. The characteristics of the line were described earlier [32].

Osteogenic, adipogenic, and chondrogenic MSC differentiation was performed as previously described [27]. Briefly, cultured MSCs (passage 5) were differentiated into the osteogenic, adipogenic, and chondrogenic lineage by culturing in the osteogenic medium (Dulbecco’s Modified Eagle’s Medium [DMEM] supplemented with 10^−8^ M dexamethasone [Sigma, St. Louis, MO, USA], 10 mM b-glycerophosphate [Sigma, USA], 50 μg/mL ascorbic acid), adipogenic medium (DMEM supplemented with 10 mM 3-isobutyl-1-methylxanthine [Sigma, USA], 0.1 mM indomethacin [Sigma, USA], 10 μg/mL insulin [Sigma, USA], 10^−6^ dexamethasone), and chondrogenic medium (Stempro, Invitrogen, Life Technologies Ltd. Paisley PA4 9RF, UK), with the subsequent staining with Alizarin Red (Sigma, USA), Oil Red O (Sigma, USA), and Alcian Blue (Sigma, USA), respectively. A Nikon Eclipse Ci microscope (Nikon Instruments Inc., New York, NY, USA) was used for the image capture.

### 2.2. Population Doubling Assay

The MSCs’ numbers were counted after 72 h of cultivation, and the doubling time was calculated. The equation for the doubling time is the following: DT = (T1 − T2) × log 2/ (log N2 − log N1), where: T1 is the initial time (hours); T2 is the final time; N1 is the initial cell number; and N2 is the final cell number [33].

### 2.3. The Plasmid and Transfection of Eukaryotic Cells

The pcNS5А-GFP plasmid encoding a chimeric complex, green fluorescent protein (GFP) fused with the full-length protein 1973–2419 aa NS5A of genotype 1b, was constructed using a commercially available pcDNA-3.1(+) vector, as previously described [34]. The GFP-NS5A chimeric complex could be detected using fluorescence microscopy without the immunocytochemical staining of the HCV gene expression product. The plasmid was prepared by purification from the *E. coli* strain *JM109*; the QIAGEN Plasmid Purification Maxi Kit (QIAGEN, Hinden, Germany) was applied in accordance with the manufacturer’s instructions.

We used seven different transfection reagents: GenJect™-39 and GenJect™-40 (Molecta, Moscow, Russia), Escort III, Escort IV, Lipofectamine 3000 (Sigma, USA), PEI (Polyethylenimine) (Invitrogen, Walthman, MA, USA), and Xfect™ Transfection Reagent (Clontech Laboratories, Inc. Takara, Mountain View, CA, USA). Transfection was performed according to the manufacturer’s instructions.

The cell culture used 24-well plates, with a total number of 5 × 10^4^ cells per well. After 24 h-long cultivation and the reaching of a subconfluent monolayer (70–90% cells/well depending on the transfection agent), complexes of the plasmid pcNS5A-GFP with the agents listed above were applied to the cells. For mice immunization, 2.5 × 10^6^ MSCs and mMSCs were seeded in several Petri dishes, with the growth area of 60.1 cm^2^. The efficiency of the transfection was assessed by GFP fluorescence after 48 h using an AxioScopeA1 inverted fluorescent microscope with an AxioCam MRc5 camera (Zeiss, Oberkochen, Germany). The percentage of fluorescent cells was calculated as the ratio of GFP-expressing cells to the total number of cells in the population.

To control the functionality of the plasmid, Huh7.5 human hepatoma cells described earlier [35] were used, and were transfected under the same conditions.

### 2.4. Sandwich ELISA for the Detection of Cytokines in the Cell Culture Medium

The measurement of the cytokine levels (IFN-α, IFN-γ, TNF-α, IL-2, IL-4, IL-6, and IL-10) was performed by ELISA in the conditioned medium from the MSCs and DP derived MSCs transfected with pcNS5A-GFP. We used the kits from Vector Best (Novosibirsk, Russia). The calibration plots for standard samples were used for the measurements of the concentrations of cytokines. The resulting values of cytokine concentrations in the conditioned medium from the MSCs were obtained by subtracting the corresponding values in the growth medium.

### 2.5. The Recombinant HCV NS5A Proteins and Synthetic Peptides

The recombinant NS5A proteins of HCV genotype 1b (aa 2061–2302, aa 2212–2313) and genotype 2a (aa 2212–2313) were purchased from the R&D Co “Diagnostic systems” (Nyzhnii Novgorod, Russia). Seven peptides representing known NS5A CTL epitopes (aa 1987-VLSDFKTWL, 2140-LLREEVSFQV, 2151-LNQYVVGSQL, 2163-EPEPDVAVL, 2225-DLIEANLLW, 2252-ILDSFDPLR, 2269-SVPAEILRK) were described previously [36]. The NS5A-specific antigens used for stimulating the T cell responses in vitro were combined into two pools: three recombinant proteins and seven peptides. The recombinant NS5A proteins were also used separately as sorbents in an enzyme-linked immunosorbent assay (ELISA) to evaluate the antibody production.

### 2.6. Immunization of Animals

Mice of the C57BL/6 (Н-2b) line (females, 6–8-week-old) were purchased from the Stolbovaya breeding and nursery laboratory (Research Center for Biomedical Technologies of FMBA, Stolbovaya, Moscow Region). All of the in vivo animal experiments were performed in accordance with Order No. 199n of the Ministry of Health of the Russian Federation and with the “Regulations on the Ethical Attitude to Laboratory Animals of N.F. Gamaleya NRCEM (Moscow, Russia)”.

To study the parameters of the immune response, we used four groups of mice with eight animals in each group. The mice from group 1 were injected with non-transfected, naïve DP-MSCs, the mice from group 2 were injected with genetically modified DP-MSCs (mMSC), the mice from group 3 were injected with the pcNS5A-GFP plasmid (Plasmid), and the mice from group 4 were injected with saline (control). MSCs and mMSCs (5 × 10^5^ cells) were injected into the tail vein, while the plasmids (100 µg) were injected intramuscularly into the quadriceps femoris muscle. Three immunizations with an interval of 2 weeks were conducted.

### 2.7. Humoral Immune Response

Eight days after the third immunization, we estimated the immune response to the administered constructs. An indirect ELISA was applied to measure the activity of antibodies against the recombinant HCV NS5A proteins in the mouse sera [24]. As secondary antibodies, antibodies against mouse Ig isotypes IgG1 and IgG2a conjugated to HRP were used (Jackson Immunoresearch Laboratories, Cambridge, UK). The reciprocal of the highest serum dilution with the optical density twice as high as that for the control group was applied as the serum titer in ELISA.

### 2.8. T-Cell Proliferation and ELISpot Assays

[^3^H]-thymidine incorporation into the DNA of dividing cells was applied for the T-cell proliferation assessment. Eight mice from each group were taken for spleen harvesting, the obtained spleens were pooled, and the splenocyte suspension was seeded in U-bottomed 96-well microculture plates with a concentration of 5 × 10^5^ cells/well. Specific stimulants were added to the splenocytes, including a pool of the seven NS5A peptides and a pool of the three recombinant NS5A proteins, with a final concentration of 1 µg/mL. As a negative control, we used the medium alone (spontaneous proliferation). All of the samples were set in at least three replicates. The cells were cultured in RPMI-1640 medium (PanEco, Moscow, Russia) containing 20% FCS (Invitrogen, San Jose, CA, USA), 4.5 mg/mL of glucose, 2 mM glutamine, 0.2 µg/mL insulin, and 50 µg/mL of gentamicin at 37 °C in a 5% CO_2_ atmosphere over a period of 5 days. The cells were labeled with 1 µCi/well [^3^H]-thymidine (provided by the Institute of Molecular Genetics of the Kurchatov Institute of the Russian Academy of Sciences, Moscow, Russia) and harvested onto the glass-fiber filters 18 h later. A MicroBeta2-counter (PerkinElmer, Waltham, MA, USA) was used for the radioactivity measurements. The results were presented in the form of stimulation indexes (SIs), defined as the ratio between the mean radioactive ^3^H incorporation as counts per minute (c.p.m.) in the presence of antigens and the mean ^3^H incorporation for the medium-only wells.

The number of cells synthesizing IFN-γ were measured with the Mouse IFN-γ ELISpotPLUS kit (HRP) (Mabtech, Stockholm, Sweden) as per the manufacturer’s instructions. An MBS-10 stereomicroscope (LZOS, Moscow Region, Russia) was utilized to visualize the stained spots. The obtained data were presented as the difference between the numbers of spots (spot-forming cells, SFC) per 10^6^ cells in the wells stimulated by the NS5A pools, and in the control stimulation-free wells that only contained the medium + 2 standard deviations (SD).

### 2.9. Flow Cytometry

The phenotype of cells from the spleen was determined using multicolor flow cytometry. The following fluorescently labeled antibodies to clusters of differentiation (CD) were used: phycoerythrin (PE)-labeled antibodies against CD11c (clone HL3) and CD8 (clone 53-6.7), fluorescein isothiocyanate (FITC)-labeled antibodies against CD4 (clone RM4-5) Gr-1 (Ly-6G and Ly-6C, clone RB6-8C5), and allophycocyanin (APC)-labeled antibodies against CD11b (clone M1/70) (BD Biosciences, San Jose, CA, USA). The proportions of naïve, effector, and memory CD4+ T cell subsets were defined by the coexpressed levels of CD62L and CD44 using the Mouse Naïve/Memory T Cell Panel (BD Pharmingen, BD Biosciences, USA). The antibodies for the corresponding isotype controls (BD 553991, BD 553930, BD 553988) were included in all of the experiments. The cells were stained as per the standard manufacturer’s protocol. A total of 10^6^ cells per probe were utilized in each case. The absolute and relative numbers of marker-bearing cells were estimated by FACS with a BD FACSCanto II flow cytometer (Beckton Dickinson, Franklin Lakes, NJ, USA) using the BD FACSDiva free software, v.6.1.3 (BD Biosciences, San Jose, CA, USA).

The flow cytometry analysis of the dental pulp-derived MSCs was performed as previously described [27]. Briefly, MSCs (passage 3–5) were washed with phosphate-buffered saline containing 1% FBS. Fluorescein isothiocyanate-conjugated anti-human CD34, CD45, and CD105 antibodies and phycoerythrin-conjugated anti-human CD29, CD44, CD73, CD90 and HLA-DR antibodies were used for staining the cells. All antibodies were purchased from Miltenyi Biotec (Bergisch Gladbach, Germany). The analysis was performed with a CyFlow Space flow cytometer (Sysmex Partec, Goerlitz, Germany) using the Partec FloMax flow cytometry Data Acquisition and Analysis software.

### 2.10. Statistical Analysis

The statistical analysis was performed with the Statistica 10 software (StatSoft Inc., Tulsa, OK, USA). GraphPad Prism (GraphpadSoftware Inc., San Diego, CA, USA, version 8.0) was used to create graphs. The data were presented as the mean ± SD or the median (minimum-maximum and interquartile range Q1–Q3) of three independent experiments. Significant differences between the groups were identified using a two-tailed Student’s *t*-test or a Mann–Whitney test as a commonly used nonparametric alternative to the *t*-test when appropriate. The multiple group comparison was performed with ANOVA followed by the Tukey test, with the *p*-value of less than 0.05 accepted as statistically significant.

## 3. Results

### 3.1. The Proliferation Activity of Dental Pulp-Derived MSCs Exceeds That of MSCs from Other Sources

**Figure 1:**
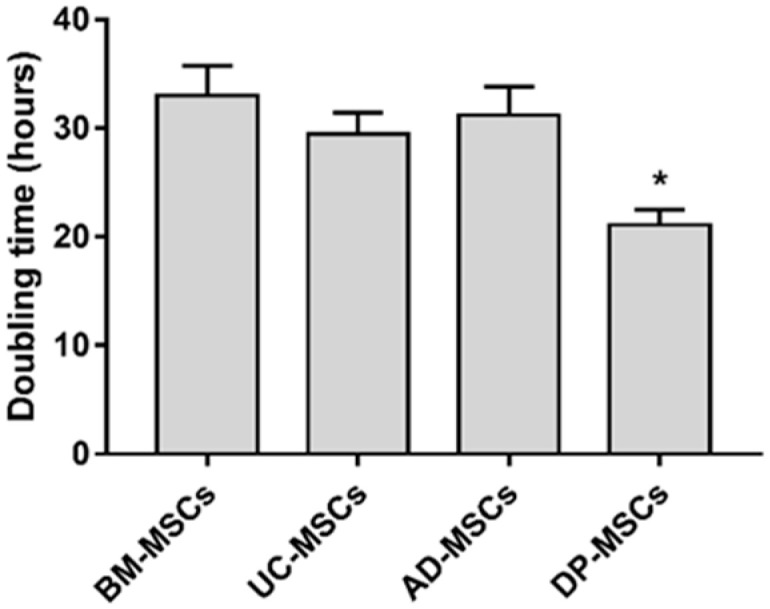
Cell doubling times for MSCs. Cell doubling times for MSCs from various sources were calculated based on the cell counts at the fourth passage. Triplicates of each group were investigated in every donor sample. The results represent the mean ± SD; * *p* < 0.05 indicates the statistical significance of DP-MSCs vs. other MSCs (One-way ANOVA and Tukey’s multiple comparison tests).

The proliferation capacity of naïve MSCs was evaluated after 72 h of cultivation (Figure 1). The data indicated that DP-MSCs showed a higher growth ability with a doubling time of 21.1 ± 1.4 h compared to BM-MSCs, UC-MSCs, and AD-MSCs, with a doubling time of one and a half times higher (*p* < 0.05).

### 3.2. Dental Pulp-Derived MSCs Characterization

**Figure 2:**
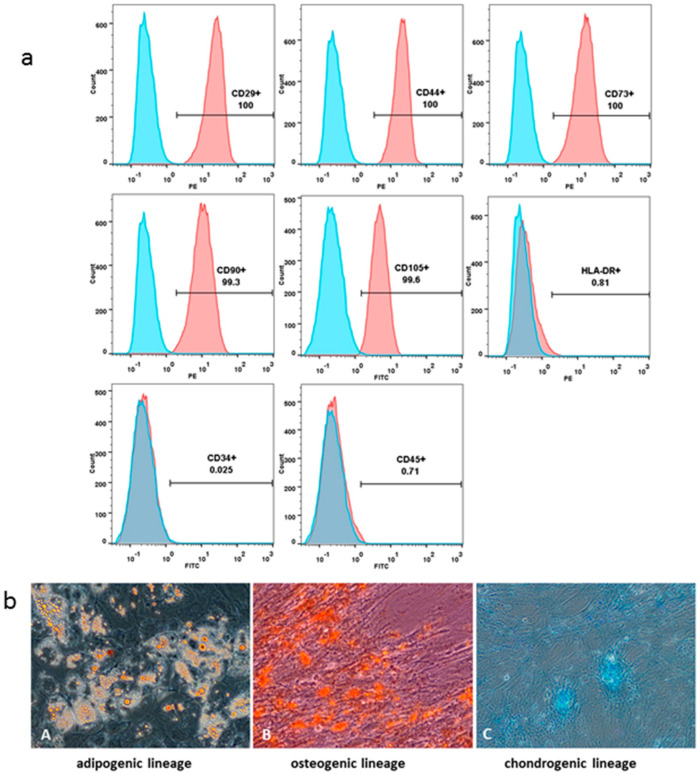
Dental pulp-derived MSCs characterization. FACS analysis of the DP-MSCs. Blue histograms represent the isotype-specific Ig control; red histograms represent FITC/PE-conjugated appropriate antibodies (**a**). MSCs’ differentiation potential analyzed by their staining with Oil Red O (adipogenic lineage, **A**), Alizarin Red (osteogenic lineage, **B**) and Alcian Blue (chondrogenic lineage, **C**) (**b**). Magnification ×200.

The FACS analysis revealed that the DP-MSCs cultured in vitro steadily expressed surface markers. DP-MSCs had a CD29+ CD44+ CD73+ CD90+ CD105+ CD34− CD45− HLA-DR− characteristic MSC phenotype (Figure 2a). Their adipogenic, osteogenic and chondrogenic differentiation potential was demonstrated by positive staining with Oil Red O, Alizarin Red, and Alcian Blue, respectively (Figure 2b).

### 3.3. Efficiency of the Gene Transfer to MSCs from Different Human Sources

**Figure 3:**
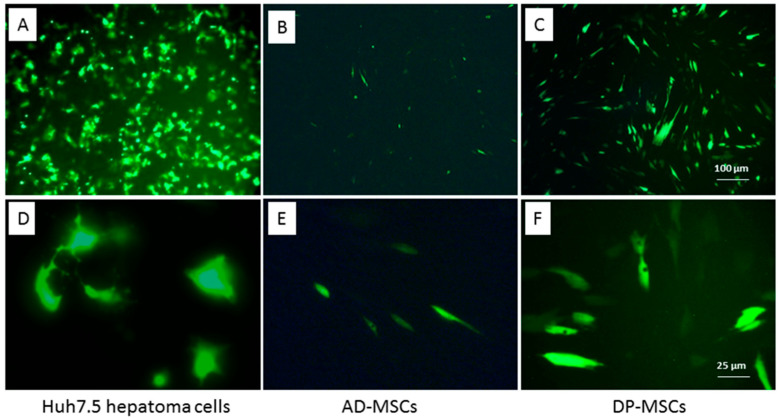
Transfection efficiency of Huh7.5 human hepatoma cells (**A**,**D**) and human MSCs from the adipose tissue (**B**,**E**) and dental pulp (**C**,**F**). GFP fluorescence 48 h after the cell transfection with the chimeric pcNS5A-GFP plasmid; scale bars, 100 µM (**A**–**C**), and 25 µM (**D**,**E**).

To obtain genetically modified MSCs (mMSCs), cells were transfected with a plasmid encoding the HCV NS5A gene and the green fluorescent protein GFP (pcNS5A-GFP). The functionality of the plasmid was confirmed by the transfection of human hepatoma Huh7.5 cells using the Xfect transfection reagent, since repeated experiments have proven the high expression of HCV proteins in these cells [35]: the number of fluorescent cells 24 h after the transfection was 30–40%, and after 48–72 h it increased to 65–80% (Figure 3A,D).

Sixteen MSC cultures derived from BM, UC, AD, and DP were transfected using different agents and transfection conditions. Cells were cultured in the growth medium supplemented with 10% FBS or HPL. Most of the MSC cultures failed transfection, or their efficiency was significantly reduced compared to that for human hepatoma tumor cells (Table 1, Figure 3B,E). A comparative analysis of the transfection efficiency with various fusion agents showed that the maximum number of GFP-positive cells was obtained using GenFect™-39. The most efficient transfection was achieved for MSCs isolated from the DP from D#15 and D#16: 48 h after the transfection, the number of cells with different luminescence intensity was 35–51% (Table 1, Figure 3C,F). The cells from D#16 were expanded in the medium containing FBS, transfected with the pcNS5A-GFP plasmid, harvested 48 h after transfection, and used for the immunization of mice.

### 3.4. The Levels of Cytokine Production Differ in Various Lines of MSCs and Change after Transfection

**Figure 4:**
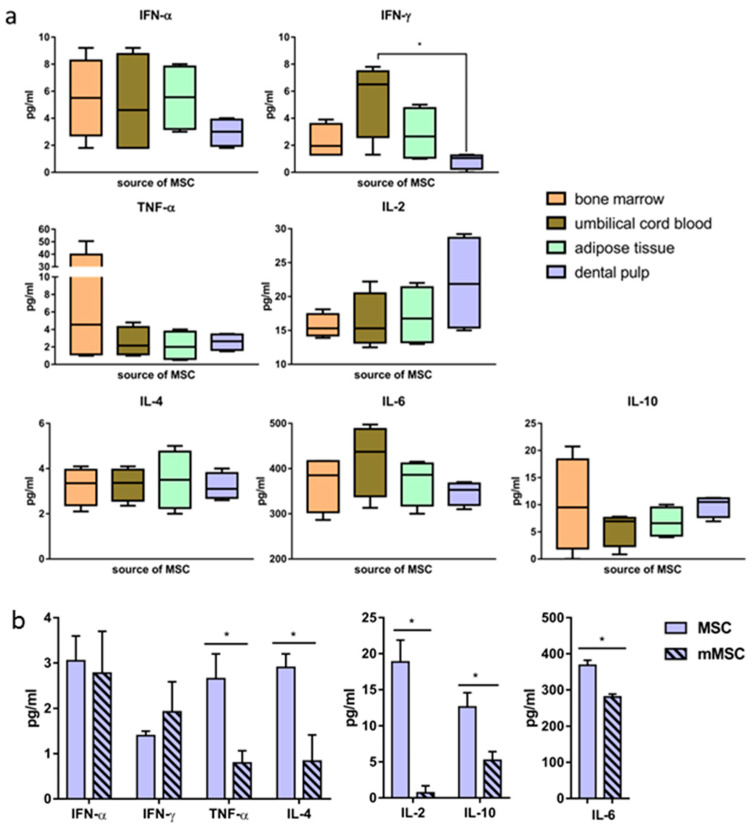
Differences in the level of cytokine secretion by human mesenchymal stem cells before and after the transfection. Concentrations of cytokines secreted by MSCs obtained from different sources; box and whisker plots (10–90% percentiles), lines at median (**a**). Changes in the concentration of cytokines secreted by dental pulp-derived naïve MSCs and mMSCs (48 h after the transfection); the values on the diagrams are the mean ± SD of three independent analyses, each of them being performed in triplicate (**b**). * *p* < 0.05 compared to the specified values.

We then measured the levels of cytokines IFN-α, IFN-γ, TNF-α, IL-2, IL-4, IL-6, and IL-10 secreted in vitro by naïve MSCs derived from the four sources. The cytokine concentrations varied widely depending on the donor from whom the MSCs were obtained, as well as on the source of the cells (Figure 4a). Statistically significant differences (*p* < 0.05) between the IFN-γ secretion by UC-MSCs and DP-MSCs were obtained. The averaged data on the concentrations of cytokines secreted into the culture medium are presented in Table 2.

It was important to define whether the pcNS5A-GFP plasmid transfection had an effect on the profile of the secreted cytokines. Forty-eight h after the transfection of DP-MSCs, the concentrations of cytokines IL-2, IL-6, TNF-α, IL-4 and IL-10 decreased statistically significantly, while the concentrations of IFN-α and IFN-γ did not change (Figure 4b).

### 3.5. The Immune Responses to Modified MSCs and to the Plasmid

The ability of human mMSCs encoding NS5A to induce an immune response to this protein was then investigated. During the in vivo experiments, neither the death of immunized mice nor their loss of body weight was recorded. The behavioral reactions in animals of the control and experimental groups did not differ. The visual assessment of the organs showed no pathological changes, which, in our opinion, indicates the safety of the injected immunogens. Three recombinant NS5A proteins were used to evaluate the humoral immune response in mice (Table S1). There were no antibodies to NS5A in the blood sera of group 1 (MSC) and 4 (Control) (titer < 1:10). In groups 2 (mMSC) and 3 (Plasmid), antibodies to NS5A of the IgG2a isotype were not detected in the sera of mice, antibodies of the IgG1 isotype were detected, but the titer did not exceed 1:20.

**Figure 5:**
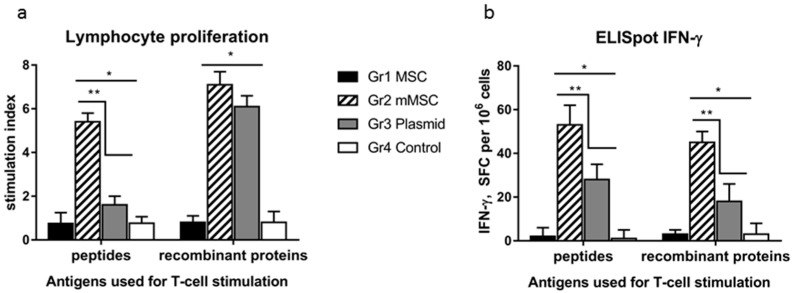
A comparative analysis of the cellular immune response in mice to the HCV NS5A protein in vitro after immunization with naïve MSC, mMSC, and the plasmid. The four groups (Gr) of mice were injected three times with non-transfected MSC (Gr1), mMSC (Gr2), the pcNS5A-GFP plasmid (Gr3), or saline (Gr4). To assess the cellular response of lymphocytes in vitro, we used the peptides and recombinant proteins from the NS5A region which were combined into two pools; the medium alone was used as a negative control. The results of T cell proliferation are expressed as stimulation indexes (SIs) (**a**); the IFN-γ production by splenocytes in response to NS5A antigens was assayed as the number of IFN-γ-synthesizing cells by ELISpot in the number of spot-forming cells (SFC) per 10^6^ cells (**b**). The values on each diagram are the mean ± SD of three measurements done in three independent experiments. * *p* < 0.05 compared to control; ** *p* < 0.05 compared to the specified groups.

The lymphocytes’ cellular response in vitro was assessed using two pools consisting of recombinant proteins and peptides from the NS5A region as specific stimulants. All the tested antigens stimulated the proliferation of splenocytes in groups 2 and 3, and the stimulation indexes (SIs) statistically significantly (*p* < 0.05) differed from the SIs in groups 1 and 4 (Figure 5a). At the same time, proliferation in response to the peptides was more than three times greater in group 2 compared to group 3, whereas it was similar in response to the recombinant proteins. 

In the ELISpot assay, the average number of IFN-γ synthesizing cells in response to the specific stimulants in groups 2 and 3 was significantly higher than that in groups 1 and 4, and in group 2 it was higher than in group 3 (Figure 5b). The differences in the signal intensity between groups 2 and 3 were 2.0 ± 0.2 and 2.5 ± 0.3 times in response to the peptides and recombinant proteins, respectively.

Thus, the immunization of mice with mMSCs expressing the NS5A gene enhances the antigen-specific lymphocyte proliferation and the number of IFN-γ-synthesizing cells. This indicates the activation of the antivirus response.

### 3.6. Increase in the Proportion of CD4+ Memory Cells in the Spleens of Mice Immunized with mMSCs

**Figure 6:**
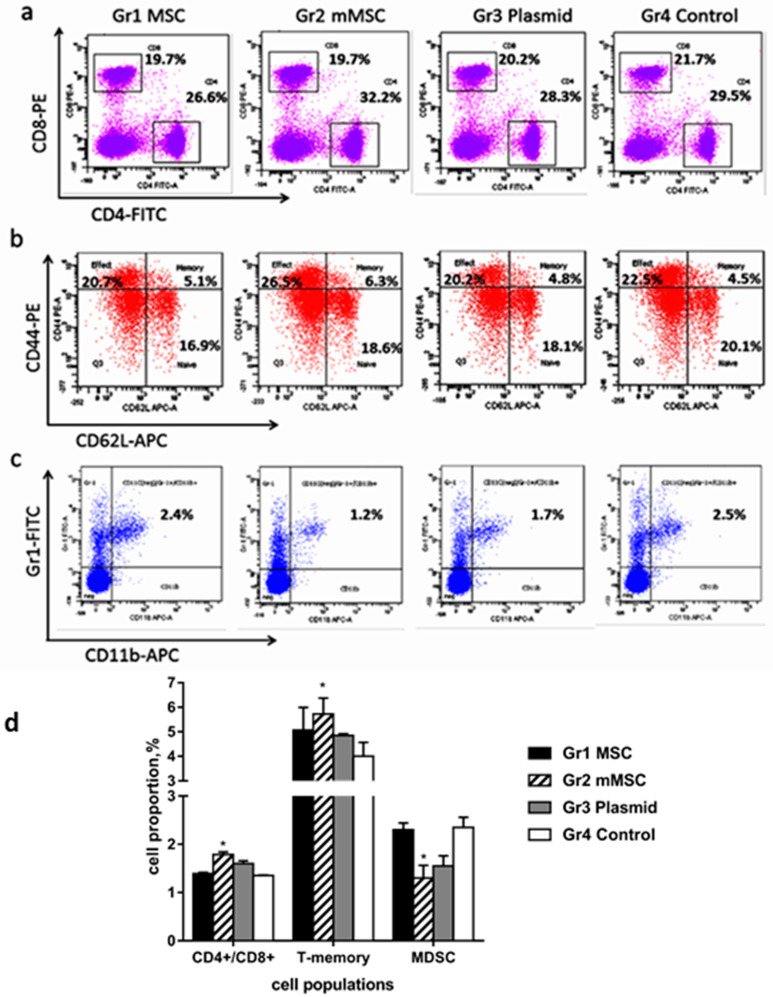
A comparative analysis of the proportion of cells in the spleens of immunized mice. Splenocytes from the immunized mice were stained with anti-CD4 and anti-CD8 antibodies (**a**) with the Mouse Naïve/Memory T Cell Panel kit (**b**) or with anti-CD11c, anti-CD11b, and anti-Gr1 antibodies (**c**), and analyzed by multicolor flow cytometry. Representative dot plots and values of the number of CD4 and CD8-positive cells are shown as percentages (**a**); representative dot plots and values of the number of CD4+ naïve (CD62^high^/CD44^low^), effector (CD62^low^/CD44^high^), and memory (CD62^high^/CD44^high^) T cells subsets are shown as percentages (**b**); representative dot plots and values of the number of MDSC expressing CD11b and Gr1, and negative for the marker of DC (CD11c) are shown as percentages (**c**); mean values of CD4+/CD8+ ratio, CD4+ T-memory cells, and MDSC are shown as percentages (**d**). The values on the diagram are the mean ± SD of three independent analyses, and each of them was performed in triplicate. * *p* < 0.05 compared to the control Gr4.

Using flow cytometry, we compared the number of splenocytes that express different receptors in immunized mice. There were no differences in the relative numbers of CD8+ (CTL) and CD4+ (Th) lymphocytes between the groups of mice (Figure 6a); however, the CD4+/CD8+ ratio was statistically significantly higher in group 2 (mMSCs, Figure 6d). The proportions of naïve, effector, and memory CD4+ T cell subsets were defined by the coexpressed levels of CD62L and CD44. The relative amount of CD4+ T-memory cells (CD62^high^/CD44^high^) increased in the spleens of mice injected with mMSCs, while the content of effector (CD62^low^/CD44^high^) and naive T-cells (CD62^high^/CD44^low^) was similar (Figure 6b,d). The content of CD11c+ dendritic cells (DC) did not differ between groups. The number of splenocytes from immunized mice that expressed CD11b+Gr-1+ markers of myeloid derived suppressor cells (MDSCs) and that did not express the CD11c+ marker of DC was evaluated (Figure 6c). A twofold decrease in the relative content of MDSCs in group 2 compared to group 4 was found (*p* < 0.05) (Figure 6c,d).

Thus, mMSCs expressing the HCV NS5A protein stimulate the formation of CD4+ memory T cells, increase the CD4+/CD8+ ratio, and reduce the content of MDSC. These are important components of an effective cellular response against HCV.

## 4. Discussion

There is little information available on obtaining and studying genetically modified MSCs as preventive and therapeutic vaccines against viral diseases. A number of studies have demonstrated the potential of mouse mMSCs as innovative vaccines to enhance the immune responses against HIV [37], hepatitis C [24,38], human papillomavirus [39], and herpes simplex virus type 1 [40]. However, the positive results obtained with mouse MSCs, when switching to experiments with human MSCs, are not always confirmed, which may be caused by differences both in the properties of the cells themselves and in the immune reactions induced by them [25,26,41]. Currently, we only aware of one work in which human mMSCs expressing the SARS-CoV-2 virus genes have been obtained, and in which the induction of virus-specific antibodies in mice immunized with mMSCs has been shown [42]. Obtaining human mMSC presents certain difficulties, since the efficiency of their transfection is significantly lower than that of mice, and varies between 2–30% depending on the fusion agent used [43,44]. In our work, most of the MSC cultures obtained from BM, including embryonic, UC, and AD showed very low transfection efficiencies of no more than 10–12%. It is assumed that foreign DNA or the toxicity of transfection reagents can lead to the activation of intracellular immune reactions in human MSCs, as well as to the suppression of transcription and translation of the transgene [45]. Both studied MSC cultures isolated from the DP were transfected significantly better: 35–50% of fluorescent cells were detected. This may be due to the higher proliferative activity of MSCs isolated from the DP (Figure 1). It is known that the efficiency of transfection directly depends on the proliferative activity of cell cultures [46]. The proportion of transfected cells reached a maximum on the second day and then decreased. According to these characteristics, the dynamics and efficiency of transgene expression in human MSCs drastically differed from those of mouse bone marrow MSCs [24]. An increase in the level of transgene expression in human cells can be achieved by transduction using viral vectors. Indeed, our pilot experiments showed a significantly higher expression of the GFP marker gene in human MSCs using recombinant adeno- and adeno-associated virus vectors vs. that with the plasmid transfection. At the same time, the efficiency of human MSCs transduction also depended on the source: it was higher in cells isolated from the DP than it was in cells from the BM. It should be noted that along with the high efficiency of viral vectors, this method of gene delivery requires solving safety problems related to the immunogenicity and insertion mutagenesis [47].

We studied, the effect of human mMSCs producing the HCV NS5A protein on the adaptive immune response of mice for the first time, and compared its quantitative characteristics with the response to a plasmid encoding the same protein and with the response to naïve, unmodified MSCs. The humoral response was weak when the NS5A sequences were injected as a part of mMSCs and the plasmid, whereas antibodies to NS5A were not detected in the control groups of mice. These data are similar to those we obtained earlier, which showed that NS5A induces a humoral immune response that is significantly less efficient compared to other non-structural HCV proteins, including NS3, NS4A/B and NS5B [24,48].

The cellular response was characterized by the lymphocyte proliferation and IFN-γ production in response to the in vitro restimulation with the HCV NS5A antigens. The cellular immune response to mMSCs significantly exceeded that of the plasmid; the differences in the signal intensity between the corresponding groups were 2.5–3 times. It should be noted that the dose of the plasmid injected into mice was 100 times higher than the dose of the same plasmid transfected in MSCs (100.0 micrograms and 1.0 microgram, respectively).

The immunization of mice with mMSCs resulted in an increase in the CD4+/CD8+ ratio. This is an important parameter of induction of the immune response associated with the increased T cell proliferation and the production of corresponding cytokines. The increased CD4+/CD8+ ratio corresponds to the increased immune function as a result of vaccination [49,50,51,52]. The importance of this parameter is evidenced by the data that patients with CHC show reduced CD4+/CD8+ ratios compared to the healthy control group, and patients with F3-F4 liver fibrosis and cirrhosis demonstrate much lower CD4+/CD8+ ratios than patients with F0-F2 fibrosis and fatty liver do [53,54,55].

It has been shown for the first time that mMSCs immunization causes an increase in the population of central T helper memory cells. The directivity of at least part of these cells to the NS5A epitopes is indirectly indicated by the absence of statistically significant differences with the control in group 1, which was administered with naïve MSCs. The fundamental difference between memory T cells and naïve T cells is that they have already undergone antigen-specific differentiation in secondary lymphoid organs. The anamnestic response mediated by long-lived memory CD4+ and CD8+ T cells is known to be faster and more aggressive than the primary response. Memory T cells are capable of rapidly acquiring the effector functions to kill infected cells and/or secrete inflammatory cytokines inhibiting the pathogen’s replication. In this regard, the formation of immunological memory is a very important property of vaccines [56,57].

The regulatory role of MSCs may be multidirectional. In cell therapy, the anti-inflammatory properties of MSCs are mainly used [18,19,20,21,58]. However, the mechanisms by which MSCs stimulate the immune response remain uncertain, and have been studied mostly in mixed leukocyte reactions in vitro [59]. The immunomodulatory effects of MSCs depend on the concentration of pro- and anti-inflammatory cytokines and other soluble factors in the microenvironment [59,60]. The high concentration of a number of cytokines in the medium, mainly IFN-γ and TNF-α, is associated with the MSC-2 phenotype and the suppression of the immune response. On the contrary, the absence of an inflammatory reaction in the body causes the polarization of MSCs into the immunostimulating phenotype of MSC-1 [61]. In this regard, a significant decrease in the secretion of TNF-α, IL-2, and IL-6 as a result of MSCs transfection may trigger their polarization into pro-inflammatory phenotypes and the activation of the immune response.

One of the mechanisms of the MSCs’ suppressor effect on the adaptive immune response in inflammation includes the inhibition of the antigen-presenting DC’s maturation related to the various MSCs-produced soluble factors—TGF-β, IL-10, NO, and PD-1 [60]. We have demonstrated here that the immunization of healthy animals did not change the DC number in all of the experimental groups compared with the control group. The data showing that immunization with mMSCs causes a twofold decrease in the number of MDSCs (a heterogeneous population of immature myeloid cells with a powerful suppressor potential) is a very interesting fact. This phenomenon for human mMSCs was observed and described for the first time. Similar data were obtained upon the immunization with mouse mMSCs carrying the NS3-NS5B genes [24]. This may indicate a common mechanism of action of mMSCs carrying the HCV genes on suppressor cells. The significance of these data becomes obvious when we note that in patients infected with HCV, an increase in the MDSC population is observed; these cells inhibit the proliferation of CD4+ and CD8+ lymphocytes, NK cells, and IFN-γ production [7,62,63]. This means that the mechanisms of MDSC suppression are involved in stimulating the innate and adaptive immune response to mMSCs in our experiments.

Thus, we consider our results as a basis for the creation of cellular vaccines against viral infections, and for the subsequent preclinical studies of the mMSCs’ protective effect in the future. Human mMSCs that express different HCV proteins, or exosomes produced by them, could be evaluated as a prophylactic agent that triggers a strong T cell response and prevents the transition of acute hepatitis C to the chronic form due to the rapid activation of memory T cells induced by the vaccine. mMSCs can also be considered as a promising therapeutic agent used in combination with DAA to increase the adaptive immunity to HCV. It can be concluded that the areas of MSC application in medical developments and in practical healthcare can be expanded significantly.

## 5. Conclusions

For the first time, the application of modified human MSCs expressing a non-structural HCV protein has been shown to be feasible for the creation of a platform for an effective anti-hepatitis C vaccine. A higher adaptive immune response induced by mMSCs was detected compared to that for the DNA immunization with the same plasmid. Human mMSCs induced more CD4+ memory T cells and led to an increase in the CD4+/CD8+ ratio. The results suggest that the immunostimulatory effect of mMSCs is associated with the switch of MSCs to the pro-inflammatory MSC-1 phenotype, as well as a reduction in the proportion of myeloid-derived suppressor cells.

## Figures and Tables

**Table 1:** Transfection efficiency of MSCs obtained from different sources.

Donor ID *	Gender	Age	MSCs Sources	Growth Medium **	Passage	Transfection Efficiency, % ***		
						24 h	48 h	72 h
D#1 [32]	-	5–6-w embryo	BM	FBS	14	2	5	2
D#2	M	21	BM	FBS	4	0	0	0
D#3	F	19	BM	FBS	3	0	0	0
D#4	M	20	BM	FBS	3	0	0	0
D#5	M	26	BM	FBS	4	0	0	0
D#6	M	19	BM	HPL	2	4	1	0
D#7	M	18	BM	HPL	3	10	4	0
D#8	F	32	UC	HPL	3	3	2	1
D#9	F	34	UC	FBS	4	0	0	0
D#10	F	30	UC	FBS	4	0	0	0
D#11	F	32	UC	HPL	2	4	12	0
D#12	F	31	UC	FBS	2	0	0	0
D#13	F	42	AD	FBS	3	1	5	1
D#14	F	65	AD	FBS	3	1	3	0
D#15	M	9	DP	FBS	3	20	35	12
D#16	M	7	DP	FBS	3–5	20	51	15

* Donor ID (denoted D#) and information for bone marrow derived (BM), umbilical cord blood derived (UC), adipose tissue derived (AD), and dental pulp derived (DP) MSCs used in transfection studies; ** Growth medium contains fetal bovine serum (FBS) or human platelet lysate (HPL); *** Transfection using GenFect™-39.

**Table 2:** Average levels of cytokines secreted into the culture medium by MSCs derived from the four sources.

	Cytokines						
	IFN-α	IFN-γ	TNF-α	IL-2	IL-6	IL-4	IL-10
Median	5.5 *	1.3	2.1	15.3	371.8	3.2	6.9
Minimum– maximum	1.8– 9.2	0– 7.8	0.5– 50.5	12.5– 29.2	286.3– 497.4	2.1– 5.0	0– 20.7
Interquartile range (Q1–Q3)	1.8– 7.4	1.3– 5.2	1.3– 4.0	15.3– 18.8	351.1– 416.3	2.9– 3.8	6.1– 9.5

* pg/mL; the resulting values of cytokine concentrations in the conditioned medium from the MSCs were obtained by subtracting the corresponding values in the growth medium.

## Data Availability

The datasets generated during and/or analyzed during the current study are available from the corresponding author upon reasonable request.

## Supplementary Materials

The following are available online at https://www.mdpi.com/article/10.3390/biology12060792/s1, Table S1: The levels of anti-NS5A antibodies of the IgG1 isotype in the sera of mice receiving three injections of MSC, mMSC, or plasmid.
