# Supplementary material for: Human Mesenchymal Stem Cells Modified with the NS5A Gene of Hepatitis C Virus Induce a Cellular Immune Response Exceeding the Response to DNA Immunization with This Gene
Source: Biology (Basel). 2023 May 30;12(6):792. doi: 10.3390/biology12060792 (PMC10295215; doi:10.3390/biology12060792)
Supplement: Supplementary file 1 [file biology-12-00792-s001.zip › biology-2394420-supplementary.pdf]

Supplementary materials for:

Article

Human mesenchymal stem cells modified with the NS5A gene of hepatitis C virus induce a cellular immune response exceeding the response to DNA immunization with this gene

Olga V. Masalova [1,*] Ekaterina I. Lesnova [1], Vladimir A. Kalsin [2], Regina R. Klimova [1], Natalya E. Fedorova [1], Vyacheslav V. Kozlov [1], Natalya A. Demidova [1], Kirill I. Yurlov [1], Mikhail A. Konoplyannikov [2,3], Tatyana N. Nikolaeva [1], Alexander V. Pronin [1], Vladimir P. Baklaushev [2] and Alla A. Kushch [1]

[1] Gamaleya National Research Center for Epidemiology and Microbiology, Ministry of Health of the Russian Federation, Moscow 123098, Russia; ol.mas@mail.ru (O.V.M.); wolf252006@yandex.ru (E.I.L.); regi.k@mail.ru (R.R.K.); ninani@mail.ru (N.E.F.); hyperslava@yandex.ru (V.V.K.); ailande@yandex.ru (N.A.D.); kir34292@yandex.ru (K.I.Y.); tatyananik.55@mail.ru (T.N.N.); proninalexander@yandex.ru (A.V.P.); vitallku@mail.ru (A.A.K.)
[2] Federal Research Clinical Center Of Specialized Medical Care and Medical Technologies, Federal Medical-Biological Agency of the Russian Federation, Moscow 115682, Russia; vkalsin@mail.ru (V.A.K.); mkonopl@mail.ru (M.A.K.); Baklaushev.vp@fnkc-fmba.ru (V.P.B.)
[3] Institute for Regenerative Medicine, Sechenov First Moscow State Medical University, Moscow 119435, Russia
* Correspondence: ol.mas@mail.ru; Tel.: +7-499-190-3049

Table S1. The levels of anti-NS5A antibodies of the IgG1 isotype in the sera of mice receiving three injections of MSC, mMSC, or plasmid.

The recombinant NS5A proteins	Gr1 MSC	Gr2 mMSC	Gr3 Plasmid	Gr4 Control
aa 2061–2302, genotype 1b	<10 (0/8)	20±5* (6/8)	20±3* (4/8)	<10 (0/8)
aa 2212–2313, genotype 1b	<10 (0/8)	15±10* (5/8)	18±7* (3/8)	<10 (0/8)
aa 2212–2313, genotype 2a	<10 (0/8)	12±5 (3/8)	10±7 (2/8)	<10 (0/8)

The four groups (Gr) of mice were injected triple with non-transfected MSC (Gr1), mMSC (Gr2), the pcNS5A-GFP plasmid (Gr3), or saline (Gr4). The recombinant NS5A proteins described in the Materials and Methods were used as sorbents in ELISA to evaluate the antibody production. The values show the geometric mean titer ± SD of three measurements. * $p < 0.05$ compared to Gr1 and Gr4. The number of mice in each group that developed antibodies to the recombinant NS5A proteins out of the total number of mice are given in the brackets.
